# Effect of a Prescription Drug Monitoring Program on Emergency Department Opioid Prescribing

**DOI:** 10.5811/westjem.2021.1.49652

**Published:** 2021-04-19

**Authors:** Rahul Gupta, Sue Boehmer, David Giampetro, Anuj Gupta, Christopher J. DeFlitch

**Affiliations:** * Penn State College of Medicine, Hershey, Pennsylvania; † Penn State Hershey Medical Center, Department of Public Health Services, Division of Biostatistics, Hershey, Pennsylvania; ‡ Penn State Hershey Medical Center, Department of Anesthesiology and Perioperative Medicine; § University of Texas at Dallas, Richardson, Texas; ¶ Penn State Hershey Medical Center, Department of Emergency Medicine, Hershey, Pennsylvania

## Abstract

**Introduction:**

Our goal was to determine whether implementation of a prescription drug monitoring program (PDMP) altered emergency department (ED) opioid prescription rates overall and in patients of different pain severities.

**Methods:**

We conducted this single-center, retrospective review at an academic ED. The study examined patients discharged from the ED who received opioid prescriptions, before and after the state’s implementation of a PDMP (August 25, 2016). The monthly rate was a ratio of the patients given ≥ 1 opioid prescription to the ED patients with a numeric pain rating scale (NPRS) > 0. We performed an interrupted time series analysis on each demographic.

**Results:**

The overall ED opioid prescription rate decreased from 51.3% (95% confidence interval [Cl], 50.4%–52.2%) to 47.9% (95% Cl, 47.0%–48.7%). For males, this decreased from 51.1% to 46.7% (P < 0.0001), while in females it did not significantly change (51.6% to 49.7% [P = 0.0529]). For those with mild pain, the rate increased from 27.5% to 34.3% (P < 0.0001), while for those with moderate pain, it did not significantly change (42.8% to 43.5% [P = 0.5924]). For those with severe pain, the rate decreased from 66.1% to 59.6% (P < 0.0001).

**Conclusion:**

We found that PDMP implementation was associated with an overall decrease in opioid prescription rates, and that patients with mild pain were prescribed opioids more often while severe pain patients were prescribed opioids less often.

## INTRODUCTION

Rates of opioid prescription and opioid-related deaths in the United States have quadrupled over the past three decades.^1^,^2^ Particularly among the elderly Medicare population, hospitalizations secondary to opioid overdose have quintupled from 1993 to 2012.^1^ Although some studies investigating opioid prescriptions in the emergency department (ED) show decreasing trends in recent years, new models expect the opioid crisis to worsen with the annual number of opioid overdose deaths projected to increase to 81,700 by 2025 from 33,100 in 2015.^3^–^5^ Given the number of opioids being prescribed by physicians, evidence also demonstrates that prescription opioid misuse can often result in the downstream use of illicit opioids, such as heroin.^6^–^10^ The concern of illicit drug use, as well as the misuse of prescription opioids, has brought the topic of monitoring the distribution of prescription drugs in the healthcare setting into the limelight.

In analyses of the distribution of opioid prescriptions, prescriptions in the ED are often carefully scrutinized.^1^,^11^ In a study that examined the rate of opioid prescribing in a single hospital, it was found that the highest rate of opioid prescribing occurred in the ED, with an opioid prescription rate of up to more than three times that of other hospital departments.^1^,^12^–^15^ Another reason that the ED serves as one of the targets for the opioid crisis is that pain is the most common chief complaint and accounts for up to 78% of visits to the ED.^16^–^19^ With such a high rate of pain complaints in the ED leading to higher rates of opioid prescribing, prescription drug monitoring programs (PDMP) have become an important tool for emergency physicians to ensure that they are not over-prescribing opioid medications and not prescribing opioid medications to patients seeking to misuse opioids.

With access to the PDMP database, emergency physicians can acquire information regarding a patient’s past controlled substance prescriptions and their frequency. Physicians can then tag individuals who are at high risk for misuse of opioid prescriptions. The primary objective of the implementation of PDMPs is to reduce the mortality and misuse of both prescription opioids and illicit opioids.^20^ Therefore, it is important to identify and analyze the regulations and legislation enacted by each state PDMP to determine the effectiveness of a PDMP implementation.^11^,^21^,^22^ In Pennsylvania, as of November 19, 2019, the PDMP shares its database with 21 other states and the District of Columbia.^23^ Access to other states through this dynamic and integrated database allows physicians to ensure that patients who cross interstate borders to fill opioid prescriptions will still be detected.^23^

Current literature examining opioid prescription rates after PDMP implementation fail to show consistent results, likely due to program variability from state to state.^24^–^29^ In addition, no studies have examined the effect of a PDMP on opioid prescription rates within pain scale cohorts using interrupted time series analyses. Therefore, our objective in this study was to determine whether a PDMP implementation alters ED opioid prescription rates overall or alters the prescription rates in patients with different pain severities.

## METHODS

### Study Design

We conducted this single-center, retrospective review at an academic, suburban, tertiary ED. The overall study examined patients discharged from the ED who received opioid prescriptions that were recorded in the electronic health record (EHR), before and after the state implemented a PDMP system. In Pennsylvania physicians are required to query the PDMP database whenever they are prescribing an opioid or benzodiazepine. They are also required to query the PDMP if they believe that the patient may be a high-risk misuser of prescription drugs. This study was approved by the institutional review board.

### Participants/Study Subjects

We retrospectively extracted data from the EHR from December 2014–May 2018. Any patient who met the inclusion/exclusion criteria was included in the study. Inclusion criteria were as follows: the patient had to have been triaged and treated by a physician in the ED during the given timeframe; prescribed at least one opioid prescription at the end of their visit; discharged directly from the ED; and to have had a rating greater than zero on the initial numeric pain rating scale (NPRS). We excluded patients if they arrived to the ED outside of the timeframe of the study, were admitted to the hospital for any period of time, had a documented NPRS of zero, did not have one of the study variables documented in the EHR, and were not prescribed an opioid at the end of their visit. A baseline patient total was also extracted from the EHR for each month of the study with the same inclusion/exclusion criteria, with the exception of a required opioid prescription. This data, which served as the denominator in our primary outcome measure, provided the total number of ED patients who presented with any level of pain, but were not necessarily given an opioid prescription at the end of their visit. In addition, if the same patient visited the ED multiple times during the study period and met inclusion criteria, they were considered as separate patients.

**Population Health Research Capsule**

What do we already know about this issue?

*Despite the seeming downtrend in opioid prescribing, concern remains for misuse.*

What was the research question?

*Does implementation of a prescription drug monitoring program (PDMP) alter ED opioid prescription rates overall and for patients with different pain severities?*

What was the major finding of the study?

*Post-PDMP, opioid prescribing decreased overall for males and for patients in severe pain, but increased for those with mild pain.*

How does this improve population health?

*Given the prevalence of opioid misuse, implementing a PDMP could potentially decrease opioid prescribing and thereby also reduce misuse.*

### Variables/Outcome Measures

We extracted the following data from the EHR: age; gender; date of encounter; and initial NPRS score (ranges 0–10). Pain scores were separated as follows: 1–4 (mild); 5–6 (moderate); and 7–10 (severe). These stratifications were based on a previous study that found these ranges to be the most accurate for each qualitative measure of pain severity based on a large-scale community survey.^30^ We compared monthly rates of opioid prescriptions by emergency physicians from December 2014–August 2016 (pre-PDMP) to the rates from September 2016–May 2018 (post-PDMP). Implementation of the PDMP in Pennsylvania occurred on August 25, 2016. The monthly rate was a ratio of ED patients meeting study criteria given ≥ 1 opioid prescription to those meeting study criteria with a NPRS > 0. The monthly rate of opioids prescribed, as a percentage, was the primary outcome measure. The monthly rate outcome measure was then analyzed overall and between different pain cohorts, genders, and age groups.

### Statistical Analysis

We performed an interrupted time series analysis to determine whether the PDMP intervention resulted in a change in the opioid prescribing rates. The periods before and after the PDMP implementation constitute the two segments of the regression model. We used the Durbin-Watson statistic to detect auto-correlation. Exploratory subgroup analyses were performed on gender, age, and pain score. Descriptive statistics were generated including means, medians, and standard deviations for continuous variables; frequency tables and odds ratios were calculated for categorical variables. We used Pearson’s chi-square test to compare overall rates before and after the PDMP implementation. The 95% confidence intervals around reported estimates reflect 0.025 in each tail, or *P* values no higher than 0.05. All analyses were performed with SAS software, version 9.4 (SAS Institute, Inc., Cary, NC).

## RESULTS

### General Characteristics

A total of 27,390 patient ED visits initially meeting study criteria were extracted from the EHR between the full time period. Of those, 587 patients were excluded by the statistician and the principal investigator due to duplicate reports of the same visit, incomplete data, or incorrectly documented data. This resulted in 26,803 patient ED visits meeting study criteria, which were then reviewed. The patients were then divided into two separate cohorts based on date of encounter: pre-PDMP (December 2014–August 2016) and post-PDMP (September 2016–May 2018). Table 1 shows the gender distribution, mean NPRS, mean age, and proportion of patients prescribed opioids overall and in the pre- and post-PDMP periods. As noted in Table 1, a majority of the patients overall, and in both the pre- and post-PDMP cohorts, identified as male and reported having severe pain. The overall mean age was 40.5 years old and the overall average NPRS was 6.2. Additionally, we found that 49.4% of patients were prescribed opioids in the selected patient population, with a decrease from 51.3% pre-PDMP to 47.9% post-PDMP.

### Monthly Rate Changes

**Figure 1:**
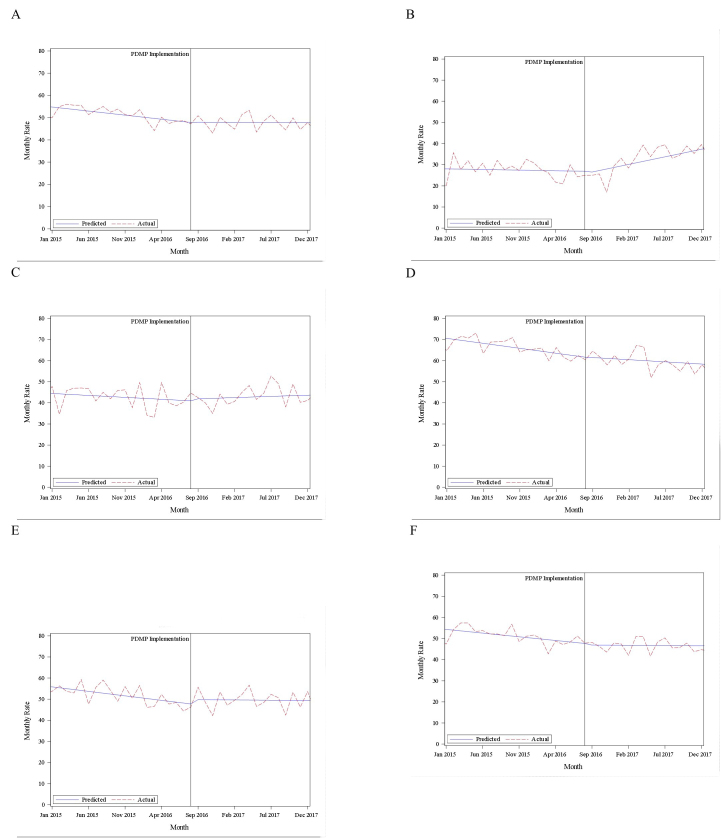
Interrupted time series analyses for A) Overall; B) mild pain (NPRS 1–4). C) moderate pain (NPRS 5–6); D) severe pain (NPRS 7–10); E) male patients; F) female patients. *NPRS*, numeric pain rating scale.

Table 2 shows the changes observed in ED opioid prescribing rates from the pre-PDMP period to the post-PDMP period in the overall cohort of patients, each gender, each NPRS cohort, and different age cohorts. Our study demonstrated a significant decrease in the opioid prescription rate from 51.3% pre-PDMP to 47.9% post-PDMP. The opioid prescription rate significantly decreased in males, but not in females. From pre-PDMP to post-PDMP, the opioid prescription rate increased significantly for mild pain patients, decreased for severe pain patients, and did not significantly change for moderate pain patients. The change in opioid prescription rate varied when compared within age groups. The results of the interrupted time series analyses based on pain cohort and gender can be seen in Figure 1.

## DISCUSSION

Some suggest the primary goal of the PDMP is to curb the opioid epidemic by allowing physicians to detect patients who may be prescribed opioids too frequently.^27^–^29^, ^31^ At the very least, a PDMP provides some transparency to the rate of opioid prescribing. In the current study, the implementation of the PDMP was associated with an overall significant decrease in opioid prescription rates in this ED. Furthermore, current literature estimates that an average opioid prescription from an ED contains about 16.6 pills.^32^ Given this data and the 3.4% decrease in opioid prescriptions after PDMP implementation in our study, it can be estimated that 501 fewer opioid prescriptions and 8322 fewer opioid pills were distributed to the community after the PDMP implementation in our ED. Therefore, our findings demonstrate an overall success in reducing opioid prescription rates that could lead to lessening the risk of subsequent opioid misuse and overdose deaths.

Ultimately, the PDMP is believed to have a multifaceted function in diminishing the opioid epidemic. Although the primary purpose of the PDMP is to curb the number of opioid prescriptions, current literature has found that the implementation of the PDMP also increases conscious awareness of physicians regarding opioid prescribing and aids in identification of patients with opioid use disorder, allowing for timely referral to interventional programs.^33^ Current literature on the effect of the PDMP on opioid prescribing trends has not shown consistent results.^24^–^29^ Maughan et al conducted a study on the effect of the PDMP on ED visits involving opioids and found there was no change in ED visits following PDMP implementation^24^. Similarly, McAllister et al and Sun et al conducted studies that found PDMP implementation did not change opioid prescribing trends in the ED.^25^,^26^ However, similar to our study, other studies found a decline in the number of opioid prescriptions in the ED following a statewide PDMP implementation.^27^–^29^

The varying trends in each study can likely be attributed to differing protocols at each institution, contrasting clinical presentations to each health system, and ultimately varying state guidelines regarding the use of the PDMP.^34^ While state variability cannot be controlled by the institution, the ease of use at each institution is often a topic of consideration when exploring how to improve the effect of the PDMP.^31^ Oftentimes, information in the PDMP is unorganized or challenging to analyze during a busy ED workflow.^35^ In addition, clinician training on how to use the PDMP also varies at each institution, often leaving busy emergency physicians with limited expertise regarding efficient use and extraction of pertinent data from the typically external PDMP.^36^ A new solution to curb some of these common challenges has been the direct point-of-care integration of the PDMP into the EHR. This solution bypasses the challenge of having a separate login mechanism on an external website and also minimizes the hindrance to ED workflow.^34^ Such an EHR integration was found to be effective as the study showed that 58% of physicians prescribed either fewer opioids or smaller quantities after the integration of the PDMP into the EHR.

In addition, we also found distinct changes in prescribing habits within different pain cohorts. With the PDMP implementation, patients experiencing mild pain were prescribed opioids more frequently while patients with severe pain were prescribed opioids less frequently. To our knowledge, no other studies have performed interrupted time series analyses after PDMP implementation in the ED on mild, moderate, and severe pain cohorts. Similarly, literature is sparse regarding the effect of a PDMP implementation on patients presenting with different pain severities. The changes we noted could have been due to the fact that patients with severe pain more often present with chronic pain and, thus, more typically already are being prescribed opioids in the PDMP. In addition, it has also been noted in current literature that drug-seeking behavior in the ED is typically associated with higher reported pain severity on the NPRS scale.^37^ Given that patients who are typically chronic users of opioids have higher pain scale ratings, it is possible that the implementation of the PDMP made this association transparent to the physician. Ultimately, our findings regarding the effect of the PDMP directly on particular pain cohorts could encourage future discussion and research.

We also found that males were significantly less likely to be prescribed opioids in the ED after PDMP implementation, while there was no change for prescribing to females. In addition, we found that all three age cohorts (18–33; 33–48; 48–63) between ages 18 and 63 years were significantly less likely to be prescribed opioids in the ED after PDMP implementation. This differs from other studies that found no differences in opioid prescribing with regard to age or gender after the implementation of a PDMP.

## LIMITATIONS

There are limitations to our study. It is important to note that it is not possible to attribute the implementation of the Pennsylvania PDMP as the sole cause for reduction of overall opioid prescribing trends. The data can only show time-dependent correlation, not causation. Given that in the current study, opioid prescription rates were already downtrending prior to the studied intervention, it is possible that other factors may have played a role, including expeditiously increasing awareness regarding the opioid epidemic and guidelines to curb it, the release of the US Centers for Disease Control and Prevention Guidelines for Opioid Prescribing in March 2016, and institutional changes that may have coincided with the implementation of the PDMP. Also, because our data relies on the accuracy of pharmacists and physicians entering data into the EHR and the PDMP, it is possible that errors occurred in the process.

The current study also relies on the fact that all patients with a complaint of pain had a pain score documented in the EHR; moreover, if a patient did not have a pain score documented, they were excluded from the study, thereby altering the accuracy of our opioid prescription rate. Also, although Pennsylvania requires a prescriber to query the PDMP before prescribing an opioid, we were not able to capture compliance rates in this study. It is important to note, however, that at the time of the study, the institution did not have PDMP data automatically imported into the patient chart. In addition, as this study was only conducted at a single hospital in Pennsylvania, it may not be easily generalizable.

While state and institutional guidelines vary, this study does provide new data regarding the effect of the PDMP on specific pain cohorts. It is also important to note that the pain scores collected were the initial pain score in the ED, which often coincided with the triage pain scores. Therefore, some of the ED treatments likely may have improved patients’ pain during their visits. Lastly, we were unable to analyze specific medications, and therefore the dosage (ie, morphine milligram equivalents) and number of pills, that were prescribed to patients in the ED. This data would have provided important information that could have aided in targeting the reduction of certain opioid prescriptions.

## CONCLUSION

Based on this analysis of opioid prescriptions pre- and post-PDMP implementation, we found that the implementation of the PDMP was associated with an overall significant decrease in opioid prescription rates in this ED. In addition, we also found that after the implementation of the PDMP, patients with mild pain were prescribed opioids more often, while those with severe pain were prescribed opioids less often.

## Figures and Tables

**Table 1:** Study characteristics of percentage of pain patients in each cohort.

	Total (n=26,803)	Number in Pre-PDMP cohort (n=12,058)	Number in Post-PDMP cohort (n=14,745)
Gender			
Males	61.1% (n=16,385)	61.4% (n=7,399)	60.9% (n=8,986)
Females	38.9% (n=10,418)	38.6% (n=4,659)	39.1% (n=5,759)
Pain scale			
Mild (1–4)	29.0% (n=7,781)	25.3% (n=3,049)	32.1% (n=4,732)
Moderate (5–6)	22.1% (n=5,922)	21.7% (n=2,621)	22.4% (n=3,301)
Severe (7–10)	48.9% (n=13,100)	53.0% (n=6,388)	45.5% (n=6,712)
Average NPRS	6.2	6.4	6.3
Mean age (yrs old)	40.5	40.7	40.4
Percent prescribed opioids	49.4% (n=13,239)	51.3% (n=6,183)	47.9% (n=7,056)

*PDMP*, prescription drug monitoring program; *yrs*, years; *NPRS*, numerical pain rating scale.

**Table 2:** Summary of changes in opioid prescription rates pre- and post-implementation of a prescription drug monitoring program.

	Pre-PDMP opioid prescription rate	95% Cl	Post-PDMP opioid prescription rate	95% Cl	% Change	P-value
Overall Rate	51.3%	(50.4, 52.2)	47.9%	(47.0, 48.7)	−3.4%	P < 0.0001
Gender						
Males	51.1%	(49.9, 52.2)	46.7%	(45.6, 47.7)	−4.4%	P < 0.0001
Females	51.6%	(50.2, 53.1)	49.7%	(48.4, 51.0)	−1.9%	P = 0.0529
Pain scale						
Mild (1–4)	27.5%	(25.9, 29.1)	34.3%	(32.9, 35.6)	6.8%	P < 0.0001
Moderate (5–6)	42.8%	(40.9, 44.7)	43.5%	(41.8, 45.2)	0.7%	P = 0.5924
Severe (7–10)	66.1%	(65.1, 67.3)	59.6%	(58.4, 60.7)	−6.5%	P < 0.0001
Age						
<18 years old	22.1%	(19.9, 24.2)	23.6%	(21.7, 25.5)	1.5%	P = 0.2946
18–33 years old	49.7%	(48.0, 51.4)	46.4%	(44.9, 48.0)	−3.3%	P = 0.0054
33–48 years old	57.4%	(55.6, 59.1)	53.2%	(51.6, 54.8)	−4.2%	P = 0.0005
48–63 years old	61.1%	(59.2, 63.1)	55.6%	(53.8, 57.4)	−5.5%	P < 0.0001
>63 years old	53.7%	(51.3, 56.1)	52.7%	(50.5, 54.9)	−1.0%	P = 0.5348

*PDMP*, prescription drug monitoring program; *CI*, confidence interval.
